# Laparoscopic conservative management of ureteral endometriosis: a survey of eighty patients submitted to ureterolysis

**DOI:** 10.1186/1477-7827-7-109

**Published:** 2009-10-12

**Authors:** Marco Camanni, Luca Bonino, Elena Maria Delpiano, Paola Berchialla, Giuseppe Migliaretti, Alberto Revelli, Francesco Deltetto

**Affiliations:** 1 GINTEAM Unit of Minimally Invasive Gynaecology, Corso Marconi 35, 10125 Turin, Italy; 2 Department of Public Health and Microbiology, University of Turin, Via Santena 5bis, 10126 Turin, Italy; 3 Reproductive Medicine and IVF Unit, Department of Obstetrical and Gynecological Sciences, Via Ventimiglia 3, 10126 Turin, Italy

## Abstract

**Background:**

this study aims to evaluate the effectiveness and safety of laparoscopic conservative management of ureteral endometriosis.

**Methods:**

Eighty cases of histologically confirmed endometriosis affecting the ureter, 10 of which with bladder involvement were prospectively studied. In detail, patients were 13 women with ureteral stenosis (7 with hydronephrosis), 32 with circular lesions totally encasing the ureter, and 35 with endometriotic foci on the ureteral wall, but not completely encasing it. They were submitted to laparoscopic ureterolysis with or without partial cystectomy, ureteroneocistostomy. The rate of surgical complications, the recurrence rate, the patients' satisfaction rate was assessed during 22 months (median) follow-up.

**Results:**

Laparoscopic ureterolysis was employed for all patients and set free the ureter from the disease in 95% of cases, whereas ureteroneocystostomy was necessary for 4 patients showing severe stenosis with hydronephrosis, among which 2 had intrinsic endometriosis of the ureteral muscularis. Three post-surgery ureteral fistulae occurred in cases with ureteral involvement longer than 4 cm: two cases were successfully treated placing double J catheter, the third needed ureteroneocistostomy. During follow-up, ureteral endometriosis recurred in 2 patients who consequently underwent ureteroneocystostomy. Most patients expressed high satisfaction rate throughout the whole follow-up period.

**Conclusion:**

laparoscopic ureterolysis is effective and well tolerated in most cases of ureteral endometriosis. Ureteroneocystostomy is a better strategy for patients with extended (more than 4 cm) ureteral involvement or with severe stenosis with or without hydronephrosis.

## Background

In the general female population, the overall prevalence of pelvic endometriosis, either superficial or deep, is estimated to be around 10% [1]. Deeply infiltrating endometriosis (DIE) may involve the urinary tract (bladder and/or ureter), sometimes causing urinary symptoms (dysuria). The urinary tract involvement by DIE is quite rare, being observed in 0,03-5% of cases [2-6]; more specifically, ureteral endometriosis is estimated to occur in 0.08%-1% of patients [6,7].

Ureteral endometriosis may be extrinsic or intrinsic: in the former case, endometriosis is located on the ureter or in close proximity to it, but does not infiltrate the ureteral muscularis; ureteral stenosis is usually less severe or even absent, with rare occurrence of hydronephrosis [2,4-6]. Differently, in case of intrinsic ureteral endometriosis, endometriotic tissue infiltrates the muscularis, stenosis is usually more severe, and hydronephrosis is more frequently associated [2,4-6]. The diagnosis of ureteral endometriosis is rather difficult because there are no symptoms or signs that are specific for this condition; in some cases, the onset of severe stenosis may lead to symptomatic hydronephrosis and finally compromise renal function [5].

The surgical management of DIE involving the ureter is a complex procedure that requires accurate balance between the need of complete excision of endometriotic foci and the need of avoiding any morbidity associated with radical surgery [5]. Due to the rarity of ureteral involvement in women with pelvic DIE, large, controlled, randomized studies on its surgical treatment are still lacking, and a clear surgical strategy to face this condition has not been identified yet. Herein we report our experience and provide information about the effectiveness and safety of our strategy of laparoscopic conservative management of ureteral endometriosis.

## Methods

### Study population

Data were collected on 808 patients undergoing operative laparoscopy for pelvic endometriosis between January 2005 and October 2007. Among these women, 80 patients had DIE affecting the ureter (and sometimes the bladder too); the endometriotic nature of the observed lesions was subsequently confirmed in all cases by histological examination of the removed tissue.

### Diagnostic workup

All patients were evaluated obtaining a detailed history and recording any symptom possibly related to urinary tract endometriosis. Each patient underwent vaginal and rectal examination, US transvaginal scan according to the technique described by Bazot [8], and transabdominal ultrasound to evaluate kidney morphology as well as the eventual presence of ureteral dilatation and/or hydronephrosis. Magnetic resonance imaging was performed only when renal alterations or hydronephrosis were signalled at ultrasound, when bowel endometriotic foci were suspected, or in case of previous surgery for DIE. Cystoscopy was performed in case of symptoms and/or US signs raising the suspect of endometriosis affecting the bladder.

### Surgical technique

IRB permission was obtained although the surgical procedures offered to patients did not differ from those currently used in our daily clinical practice. The patients signed a detailed informed consent illustrating the diagnostic and therapeutic workout. All surgical operations were performed by the same team, using standardized techniques.

Pre-surgical preparation began 24 hours prior to operation and consisted of oral intake of Phosfo-Lax (Sofar, Trezzano Rosa, Milan, Italy) or Selg-Esse 1000 (Promefarm, Milan, Italy), plus Mylicon tablets (Warner Lambert, Milan, Italy). Foley catheter was always inserted into the bladder at the time of surgical intervention; in most cases it was removed the day following surgery, but in case of bladder resection it was left for ten days.

All patients underwent laparoscopic ureterolysis under general anaesthesia, with standard technique including the use of 10-mm operative laparoscope and three 5-mm ancillary trocars in the presence of 12-mm Hg intra-abdominal pressure.

The first surgical step consisted in opening the peritoneum and submitting the ureter to careful blunt dissection. The dissection started where the ureter was clearly visible and without adhesions and progressed in the direction of the uterosacral ligaments until insertion into the bladder. At the end of the dissection, the ureter had to be completely mobilized and visible from the pelvic brim to its insertion into the bladder.

When endometriotic nodules were seen on the bladder wall, partial cystectomy was performed with monopolar scissors and the bladder was then closed by one-layer suture (intracorporeal knot technique). Endometriotic lesions on the ureter, both those circularly encasing it and those involving just part of its circumference, were carefully removed, regardless the presence of stenosis and/or hydronephrosis.

Ureteroneocystostomy was performed only in 4 cases of very severe ureteral stenosis with hydronephrosis, two of which resulted to be due to intrinsic endometriosis.

Endometriotic lesions at other locations were also removed. In case of recto-vaginal endometriosis without bowel involvement, a space between the ureter and the utero-sacral ligament was created, recto-vaginal septum was opened and the anterior wall of the rectum was set free from any adherence. In case of bowel involvement, the recto-vaginal septum was prepared by bilateral dissection of the pararectal space medially to the utero-sacral ligaments, and segmental bowel resection was performed.

Post-operative antibiotic treatment was not scheduled routinely, but it was administered in case of bowel resection, bladder resection, or ureteroneocystostomy.

### Follow up

All operated patients were contacted via phone both one week and one month after surgery to assess subjective well-being. Six, 12 and 24 months after surgery, each patient underwent clinical examination, transvaginal and transabdominal US scanning. At the same times, patients were also requested to answer to a specific questionnaire dealing with their symptoms and their satisfaction about surgery outcome. Patients' satisfaction was scored from 0 to10; a score of 0-4 was classified as 'dissatisfied', 5-8 as 'satisfied', and 9-10 as 'very satisfied'.

### Statistics

The SPSS (Statistical Package for the Social Sciences) version 11.0 for Windows (SPSS, Inc. in Chicago) was used for statistical analyses, that were accomplished by an independent statistician.

The prevalence of symptoms pre- and post-surgery was compared using Wilcoxon test for paired data. Post-surgery patient's satisfaction scores at 6, 12 and 24 months were compared by the Friedman test for repeated measures. P-values < 0.05 were considered statistically significant.

In order to estimate the probability of undergoing a new surgical intervention due to disease recurrence, the time lapse between primary surgery and re-intervention was analysed applying the Kaplan Meier curve at 36-months time interval.

## Results

Dysuria affected 27% of the 808 patients undergoing laparoscopic surgery for pelvic endometriosis. Laparoscopy evidenced ureteral involvement in 80/808 patients (9.9%), that were subdivided into three subgroups according to Frenna [6]: a) patients with endometriotic lesions causing severe ureteral stenosis (n = 13, among which 7 with associated hydronephrosis); b) patients with endometriotic tissue surrounding circularly and encasing the ureter but not causing severe stenosis (n = 32), and c) patients with endometriotic tissue on the ureteral wall but not encasing the organ (n = 35). The mean diameter of ureteral endometriotic lesions was 2.2 cm (range 1-6 cm). The left side was interested in 38 cases, the right in 34, both sides in 8. The bladder was involved together with the ureter in 10 cases, with endometriotic foci of 2.2 cm mean diameter (range 1-4 cm). According to the rAFS classification, in the 80 patients with ureteral endometriosis the disease was at stage IV in 45% of cases, at stage III in 30%, at stage II in 19%, and at stage I in 6% of patients.

Laparoscopic ureterolysis was performed as first surgical approach in all patients (Table 1). At the end of such procedure, in 76 cases out of 80 (95%) the ureter was completely free of endometriosis; in 4 patients with severe ureteral stenosis and hydronephrosis, ureteroneocystostomy was performed after ureterolysis during the same anaesthesia (Table 1). Considering only cases with ureteral stenosis or encased organ without stenosis (subgroups "a" and "b", n = 45), laparoscopic conservative approach was sufficient in 91% of cases (41 out of 45).

**Table 1:** Surgical procedures in 80 patients with DIE involving the ureter

	**Patients**	
	**N**	**%**
Ureterolysis	80/80	100
Ureteroneocystostomy	4/80	5
Partial cystectomy	10/80	12.5
Excision of ovarian endometriomas	54/80	67.5
Dissection of rectovaginal nodules	41/80	51.2
Dissection of vaginal nodules	7/80	8.7
Monolateral salpingectomy	3/80	3.7
Salpingo-oophorectomy	3/80	3.7
Hysterectomy	1/80	1.2
Bowel segmental resection	10/80	12.5

All cases of bladder involvement (n = 10) required partial laparoscopic cystectomy (Table 1). Other endometriotic nodules that were found intra-operatively at other locations were removed (for details see Table 1). Eight patients needed intraoperatory insertion of double J catheter, that was then removed after three months.

The endometriotic nature of ureteral lesions was histologically confirmed in all excised tissues; in 2 of the 4 cases who underwent ureteroneocystostomy, intrinsic endometriosis involving the muscularis and the uroepithelial layer was observed.

The overall time needed for operation ranged from 50 to 750 minutes, with a median of 167 minutes. Post-operative course was uneventful in all cases. The median hospitalization time was 44.6 hrs (range 20-168). None of the patients complained of post-operatory urinary retention.

The median follow-up time was 22 months (range 6-41). Three long-term surgical complications were observed out of 80 interventions (3.7%) at 10 days, 6 and 8 weeks after surgery, respectively: two patients had ureteral fistula and another patient had a fistula between left ureter, rectum and vagina. These three patients were affected by ureteral lesions extending for more than 4 cm in length, and their excision involved a long tract of the ureteral wall. In the first two patients, a few weeks with double J catheter led to complete recovery without the need of further invasive surgery. The third patient underwent bilateral extended ureterolysis, segmental bowel resection, and excision of a portion of the vagina in the first operation; during corrective surgery for the fistula via laparotomic approach, it was necessary to perform a second bowel resection together with ureteroneocystostomy.

The intensity of symptoms was significantly lower 6 months after surgery (Table 2), when all patients were asymptomatic and had normal urinary tract and renal morphology at US. Most patients showed high satisfaction rates at 6, 12 and 24 months; interestingly, this variable did not significantly decrease with time (Table 3).

**Table 2:** Pre- and post-operative (6 months) score of symptoms intensity in 80 patients with ureteral endometriosis submitted to ureterolysis

	**Pre-operative**			**Post-operative**			
	**25th**	**median**	**75th**	**25th**	**median**	**75th**	**p**
Dysmenorrhea	6	8	9	0	0	4	p < 0.001^a^
Dysuria	5	7	8	0	0	0	p < 0.001^a^
Dyschezia	5	7	8	0	0	0	p < 0.001^a^
Dyspareunia	4	6	8	0	0	0	p < 0.001^a^

^a^Wilcoxon test

**Table 3:** Degree of satisfaction after 6, 12 and 24 months from surgery in 80 patients with ureteral endometriosis submitted to ureterolysis

	**Follow-up**		
	**6 months**	**12 months**	**24 months**
	**n = 80**	**n = 64**	**n = 19**
Very satisfied	66%	70%	69%
Satisfied	32%	28%	15.5%
Not satisfied	2%	2%	15.5%

6 months vs. 12 vs. 24: p = 0.368 at Friedman test

**Figure 1:**
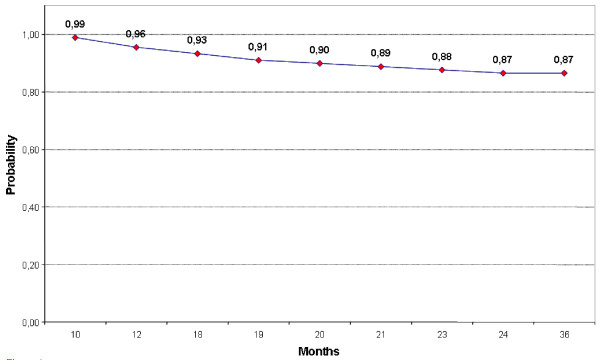
**Kaplan Meier curve referring to the time interval between primary surgery and re-operation for disease recurrence**. The line represents the probability of being disease-free at each follow-up time.

Seven patients out of 80 (8.7%) had recurrence of pelvic endometriosis after a median time of 18 months (range 7 - 24). Two of them (representing 4.4% of the 45 patients with complete circular ureteral involvement) showed recurrence of ureteral endometriosis with stenosis and were submitted to ureteroneocystostomy. Overall, according to Kaplan Meier curve the chance of not needing re-intervention during the follow-up period was 96% at 12 months and 87% at 24 months (Figure 1).

## Discussion

The management of DIE involving the ureter is aimed both at getting relief from symptoms and preserving ureteral patency and renal function. Ureteral endometriosis is quite rate, and for this reason the optimal treatment strategy to treat it has not been identified yet, the debate being still ongoing [9]. Medical therapy is considered unsufficient to resolve DIE with ureteral localisation [10-13], laparoscopic surgery may be conservative, or include ureterotomy and ureteroneocistostomy. The choice between conservative and aggressive surgical approach depends more on the surgeon's personal opinion than on scientific evidence. In fact, ureteral endometriosis is quite rate and the effectiveness of its treatment by laparoscopic surgery has not been studied in large prospective trials, but only in small surgical series with relatively short follow-up periods.

In our study, one of the largest prospective series available to date, laparoscopic ureterolysis was used as the first approach in all patients with ureteral endometriosis, both those with complete circular ureteral involvement with or without stenosis (n = 45) and those with endometriotic nodules located on the ureter wall but not encasing it (n = 35). This first-line surgical strategy was effective in 95% of patients, who had low recurrence rate and high satisfaction rates throughout the whole follow-up. Only 4 out of 80 cases, those having very severe ureteral stenosis with hydronephrosis, could not be resolved by ureterolysis and needed to be treated more invasively (ureteroneocystostomy).

Our results are consistent with previous reports that support the effectiveness of conservative laparoscopic strategy for the treatment of ureteral endometriosis [6,14-17]. The possibility of treating endometriosis-linked ureteral stenosis by laparoscopy was previously reported by Frenna [6] and some others, who described the use of laparoscopy instead of laparotomic urological surgery procedures in this pathological condition. Nezhat et al. [14] reported the resolution of ureteral obstruction in a series of 21 patients with severe ureteral endometriosis, ten of which were operated with laparoscopic ureterolysis. Similar data were reported by Donnez et al. [15], who treated laparoscopically 16 out of 18 patients with ureteral endometriosis and hydronephrosis. Seracchioli et al. [16] successfully performed laparoscopic ureterolysis in 22 cases out of 30 with ureteral endometriosis, whereas 8 patients were treated either with laparotomic uretero-ureterostomy or ureteroneocystostomy.

Considering only patients with complete circular involvement encasing the ureter, with or without stenosis, the recurrence rate in our series was 4.4%, as 2 out of 45 patients showed ureteral DIE recurrence a few months after conservative treatment. Ghezzi et al [17] reported a higher recurrence rate (12.1%) in a series of 32 patients with severe ureteral stenosis and hydronephrosis operated by laparoscopic ureterolysis; in his study, however, the considerable number of relapses is likely related to the severity of ureteral involvement and to the presence of some cases of intrinsic ureteral disease. Ghezzi's report suggests to limit the use of conservative approach to patients with lower recurrence risk. This opinion is supported by Mereu et al. [18], who prospectively studied patients with ureteral endometriosis and hydronephrosis and underlined the importance of considering ureterolysis only for milder cases of non-obstructive ureteral disease, chosing a more aggressive approach in case of obstructive ureteral involvement. In a recent series of 29 patients with severe ureteral DIE with stenosis that were operated using radical surgery, intrinsic endometriosis was observed in about half of the patients, a higher prevalence than expected [19]; since intrinsic endometriosis is associated with a very high risk of recurrence in case of incomplete eradication, ureteroneocystostomy appears to be the surgery of choice in patients with severe ureteral stenosis.

There are only a few data about the incidence of ureteral injuries during laparoscopic surgery for DIE, and the occurrence of ureteral fistulae does not appear to be linked to the severity of ureteral involvement [20-22]. Frenna reported one case of uretero-vaginal fistula in his series of 54 ureterolysis [6]. The most recent report by Mereu et al. [18] documented one case out of 56 laparoscopic operations. In our series, we observed three cases of ureteral fistula, that occurred in patients with an extensive (more than 4 cm) involvement of the ureter by endometriotic lesions, a condition that forced the surgeon to weaken a long tract of the ureteral wall. In order to limit the risk of appearance of an ureteral fistula it could be an option to leave a double J for several weeks. However, this was not our choice for the following reasons: (a) the overall incidence of fistulae is very low after ureterolysis; (b) a fistula may occur even with an in situ doubleJ catheter if the ureter undergoes significant devascularization; (c) the presence of a double J catheter would imply a long lasting antibiotic therapy and then a small intervention to remove the catheter: both would probably have a negative impact on the patient's compliance to treatment.

When endometriosis involved also the bladder wall (10 cases in our series), the association of ureterolysis to laparoscopic partial cistectomy led to long-term relief of urinary symptoms, with no increase of the recurrence rate and no major surgical complications. Such results are consistent with previous reports [14,23-28] that showed that laparoscopic partial cystectomy is effective and safe, it is less invasive than the laparotomic approach [26] and has long-lasting therapeutic effectiveness [27].

In conclusion, our findings and the data available in the medical literature confirm that ureterolysis may be used as the initial treatment option in most patients with ureteral endometriosis, eventually associating partial cistectomy and excision of other endometriotic foci in the pelvis. Ureterolysis may be employed even in selected cases of mild ureteral stenosis or when the organ is encased by endometriosis, provided that the extension of ureteral involvement is limited in length. The conservative laparoscopic approach is very well tolerated and has a reasonable incidence of complications as well as a low recurrence rate. For patients dysplaying an extended, severe ureteral involvement with severe stenosis and hydronephrosis, those with high risk of having intrinsic ureteral disease, ureterolysis is insufficient and ureteroneocystostomy represents a wiser surgical strategy.

## Competing interests

There is no conflict of interest that would prejudice the impartiality of this scientific work. This research did not receive any specific grant from any funding source in the public, commercial or not-for-profit sector.

## Authors' contributions

MC, LB, EMD and FD performed laparoscopic surgery, PB and GM contributed to writing the manuscript, AR critically reviewed the manuscript. All authors read and approved the final manuscript.
